# Assessment of Stage Two Hypertension Treatment Plans Written by Generative AI

**DOI:** 10.3390/jcm15083103

**Published:** 2026-04-18

**Authors:** Tai Metzger, Zaheen Hossain, Kody Park, Stephen Vu, Simon Dixon, Tracey A. H. Taylor

**Affiliations:** 1 Department of Foundational Medical Studies, Oakland University William Beaumont School of Medicine, Rochester, MI 48309, USA; 2 Department of Cardiovascular Medicine, Corewell Health William Beaumont University Hospital, Royal Oak, MI 48073, USA

**Keywords:** artificial intelligence, hypertension, large language models, cardiovascular disease

## Abstract

**Background/Objectives**: As use of large language models (LLMs) in clinical practice, in medical education, and by patients increases, it is essential to ensure that information provided is accurate and safe. Our objective was to compare stage two hypertension treatment plans generated by popular LLMs. **Methods**: ChatGPT (GPT-4o), Claude (Claude 4 Sonnet), ClinicalKey AI, Microsoft Copilot (Wave 2), DeepSeek-V3-0324, Dyna AI, Google Gemini (2.5 Flash), Grok (version 3), Meta AI assistant (Llama 4 Maverick), OpenEvidence (version 2.0), Perplexity (Sonar backend model), and Pi (Inflection-2.5) were prompted to generate a treatment plan for stage two hypertension. Six blinded reviewers scored each response in three domains: adherence to clinical guidelines, detail/clarity, and reliability/safety. **Results**: Perplexity received the highest composite score (8.17 out of 9), followed by OpenEvidence (7.92 out of 9). Dyna AI had the lowest overall score (3.75 out of 9). Perplexity (3.00 out of 3), Grok (2.83 out of 3), and OpenEvidence (2.75 out of 3) had the highest scores for detail/clarity, while Dyna AI had the lowest for both detail/clarity (1.00 out of 3) and reliability/safety (1.00 out of 3). ChatGPT had the highest score for adherence to guidelines (2.75 out of 3) while Pi had the lowest (1.58 out of 3). Kruskal–Wallis test showed *p* < 0.05 across sub-score domains and composite scores. **Conclusions**: LLMs tended to adhere to clinical guidelines and provide detailed responses but often did not provide sources or instruct users to see a healthcare professional. There was notable variability in quality, and medicine-specific LLMs were not superior to popular LLMs.

## 1. Introduction

The integration of artificial intelligence (AI) into healthcare has accelerated rapidly in recent years, transforming clinical practice, medical education, and patient access to health information. Clinicians are increasingly leveraging AI tools to support diagnostic accuracy, personalize treatment plans, and streamline decision-making processes. AI-driven clinical decision support systems enhance clinicians’ decisions and patient outcomes [1]. This growing reliance on AI underscores the need for robust, evidence-based systems that can augment human expertise without compromising patient safety.

In medical education, AI has emerged as a transformative tool for both students and educators. Generative AI technologies are being utilized to generate medical exam questions, produce clinical scripts for diseases, and improve the diagnostic and clinical skills of students and clinicians [2]. These applications not only enhance learning experiences but also prepare future healthcare professionals for an increasingly digital healthcare environment.

Patients are also turning to AI-powered platforms to better understand their health conditions and explore treatment options. AI tools can significantly enhance patient health literacy by making complex medical information more accessible, personalized, and interactive, thus empowering patients to take a more active role in managing their healthcare [3]. However, the proliferation of AI in patient education also raises concerns about the accuracy and reliability of the information provided, highlighting the need for careful evaluation and oversight.

Several large language models (LLMs) have gained prominence in medical and non-medical fields, each offering unique capabilities and applications. ChatGPT, developed by OpenAI, is widely used for its conversational abilities and has been explored for various medical applications. Perplexity AI and OpenEvidence provide real-time synthesis and access to medical literature, aiding medical students during clinical rotations [4]. DeepSeek, an open-source multimodal LLM, has shown potential in clinical workflows and medical education [5]. Claude, ChatGPT, Copilot, and Google’s Gemini have been evaluated for their medical knowledge in various settings [6,7]. OpenEvidence and ClinicalKey AI have been studied in the context of aiding primary care physicians in clinical settings [8].

Emerging evidence suggests that these systems can demonstrate meaningful medical knowledge and may assist clinicians in diagnostic reasoning, evidence synthesis, and clinical documentation tasks [9]. However, the clinical deployment of LLMs remains controversial because their outputs can vary substantially in accuracy and reliability. One major limitation is the phenomenon of “AI hallucinations,” in which models generate plausible-sounding but factually incorrect information with high confidence, potentially leading to misleading medical advice or incorrect clinical reasoning [10]. These risks are particularly concerning in healthcare settings, where incorrect information may affect diagnosis, treatment decisions, or patient safety [11]. Additionally, the rapid proliferation of consumer-facing AI systems has outpaced regulatory oversight, and many widely used tools are not designed or approved as medical devices despite being used for health-related queries [12]. As a result, researchers and policy experts have emphasized the need for rigorous evaluation, transparency, and regulatory frameworks to ensure that AI systems deployed in healthcare meet appropriate standards of safety, reliability, and accountability [13].

AI chatbots can be divided into three major types of structures often conflated under the label “LLM,” including pure generative models (ChatGPT, Claude, Gemini, and others), retrieval-augmented generation (RAG) systems that query external databases before synthesizing a response (Perplexity, OpenEvidence, ClinicalKey AI, Dyna AI), and a hybrid architecture (Microsoft Copilot) [14]. Notably, RAG systems inherently cite the sources of information that they draw upon whereas pure language models do not have this function.

Ensuring the accuracy of these LLMs is paramount, given their influence on medical education, clinical practice, and patient decision-making. Inaccurate or misleading information can have serious consequences, including misdiagnosis, inappropriate treatment, and erosion of trust in healthcare systems. Therefore, rigorous evaluation of these models’ performances, particularly in generating treatment recommendations, is essential to ensure they meet the high standards required in healthcare settings. In particular, information related to common chronic diseases, such as hypertension, should be among the areas most stringently examined for reliability.

Hypertension is a pervasive health issue, affecting approximately 31.1% of adults worldwide, with higher prevalence in low- and middle-income countries. It is a leading risk factor for cardiovascular diseases, including stroke and coronary heart disease, and is responsible for millions of deaths annually [15]. Despite its significant impact, hypertension often remains undiagnosed and inadequately managed, contributing to a substantial burden on global health systems [16]. Effective management of hypertension requires adherence to clinical guidelines and personalized treatment strategies [17]. Guideline-mediated management has been shown to improve blood pressure control and reduce the risk of cardiovascular events. Tailoring treatment plans to individual patient profiles, such as their comorbidities and lifestyle, enhances the efficacy of interventions and patient adherence. The integration of AI into this process offers the potential to support clinicians in developing optimized, patient-specific treatment plans [18].

This study aims to assess and compare the quality of hypertension treatment plans generated by various freely available (or with free-trial) AI-powered chatbots, including ChatGPT (OpenAI), Claude (Anthropic), ClinicalKey AI (Elsevier), Copilot (Microsoft), DeepSeek-V3, Dyna AI, Google Gemini (2.5 Flash), Grok (X), Meta AI assistant, OpenEvidence, Perplexity, and Pi. By evaluating the accuracy, completeness, and adherence to clinical guidelines of these AI-generated plans, we seek to identify the strengths and limitations of each platform. Our goal is to inform the development and implementation of AI tools in hypertension management, ensuring they effectively support clinicians, educators, and patients in achieving optimal health outcomes.

## 2. Materials and Methods

Publicly available AI chatbots were prompted to generate a hypertension treatment plan. Six blinded reviewers rated each response on adherence to clinical guidelines, thoroughness/detail, and emphasis on seeing a healthcare professional. Reviewers consisted of medical students and medical school faculty, including cardiology faculty. The 2017 American College of Cardiology (ACC)/American Heart Association (AHA) joint committee clinical practice guidelines were used as the standard for comparison in this study [17].

The prompt given to each AI chatbot was “Pretend you are a physician seeing a 50-year-old male patient who has just been diagnosed with stage 2 hypertension. What treatment plan would you provide for this patient?” and the LLM response was pasted into a document for review. Each of the twelve systems was prompted between 5 June and 12 June 2025, with most queries performed on 5 June 2025, to generate a treatment plan for stage two hypertension. The evaluated models included ChatGPT (GPT-4o, San Francisco, CA, USA), Claude (Claude 4 Sonnet, San Francisco, CA, USA), ClinicalKey AI (Amsterdam, The Netherlands), Microsoft Copilot (Wave 2, Redmond, WA, USA), DeepSeek (V3-0324, Hangzhou, China), Dyna AI (Ipswich, MA, USA), Google Gemini (2.5 Flash, Mountain View, CA, USA), Grok (version 3, Palo Alto, CA, USA), Meta AI assistant (Llama 4 Maverick, Menlo Park, CA, USA), OpenEvidence (version 2.0, Miami, FL, USA), Perplexity (Sonar backend model, San Francisco, CA, USA), and Pi (Inflection-2.5, Palo Alto, CA, USA). All systems were accessed using free-tier accounts, except for ClinicalKey AI and Dyna AI which were accessed through available free trial versions. The responses generated by each LLM were collected and compiled for blinded review. When applicable, models that provided citations or retrieved external sources were recorded. See Supplementary Material for details.

Each response was read by six reviewers and rated from 1 to 3 across three domains according to the following rubric, developed by the authors:

- Adherence to ACC/AHA clinical guidelines [17]:
  ○ 1 point: Not consistent with guidelines.
  ○ 2 points: Somewhat followed guidelines.
  ○ 3 points: Completely followed the guideline.
- Thoroughness/detail/clarity:
  ○ 1 point: Very little detail provided.
  ○ 2 points: Plan was detailed but not thorough enough to treat an actual patient.
  ○ 3 points: Plan was sufficiently detailed for care of a similar patient in real-life.
- Reliability and safety: emphasis on seeing a healthcare professional/provided credible source(s):
  ○ 1 point: Did not provide reliable source(s) or refer user to healthcare professional.
  ○ 2 points: Provided reliable source(s) or referred user to healthcare professional for medical advice (not both).
  ○ 3 points: Provided both reliable sources(s) and referred user to healthcare professional for medical advice.

Reviewers were blinded to which LLM generated each response, and all formatting was made consistent to prevent reviewers from guessing which LLM had generated the response. All responses were uploaded to a Word document and lightly edited to have the same font and text size. Any explicit references to the chatbot being used were redacted. Other metadata such as specific standard phrases and AI-specific expressions were not removed as long as they did not mention the chatbot being used. All prompts and responses were in English. Mean scores for each domain and composite scores were calculated for each LLM and compared.

All prompts were given to the LLMs without prior interactions with the chat in order to obtain responses independent of user variability, since LLMs often adjust their responses based on prior interactions. All responses were acquired on the same computer and browser (Google Chrome). Run dates and model version identifiers are included in the Supplemental Material. Additionally, it is important to note that the LLMs often generate different responses even when given the same prompt, so the responses provided here may be different from those generated by others.

To evaluate whether reviewer-assigned scores differed across large language models (LLMs), we used the Kruskal–Wallis rank-sum test, with LLM as the categorical independent variable and the reviewer score as the dependent variable. This nonparametric test was performed separately for each rubric domain (guideline adherence, detail/clarity, reliability/safety) and for the composite score. The Kruskal–Wallis test evaluates the null hypothesis that the distribution of scores is the same across all LLM groups; a statistically significant result indicates that at least one LLM differs from the others. Results were summarized as mean scores by LLM for descriptive purposes. Statistical significance was defined a priori as a two-sided *p* < 0.05, and *p* values were reported for each domain and the composite score. We calculated the Intraclass Correlation Coefficient (ICC) using a single-rater, two-way random-effects intraclass correlation coefficient (ICC [1,2]) for absolute agreement.

## 3. Results

Inter-rater reliability among the six reviewers was assessed using the Shrout–Fleiss intraclass correlation coefficient (ICC). The ICC for adherence to clinical guidelines was 0.42, for detail and clarity 0.56, and for the reliability/safety domain 0.46. The overall composite score demonstrated an ICC of 0.59, indicating moderate agreement among reviewers across the evaluated large language model responses.

**Figure 1:**
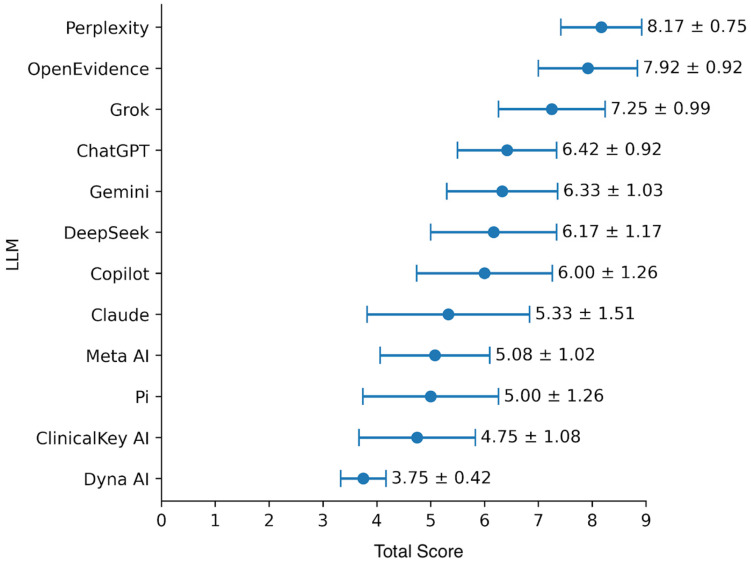
Overall HTN treatment plan scores across popular LLMs. Each LLM was assigned a score out of 9 using the rubric described in the Methods. The three sub- scores were combined for a total mean score out of 9. Error bars represent standard deviations. Kruskal–Wallis test confirmed statistical significance (χ^2^(11) = 44.2, *p* < 0.0001).

Across the twelve large language models evaluated, Perplexity achieved the highest overall hypertension (HTN) treatment plan score at 8.17 out of 9, followed by OpenEvidence at 7.92 and Grok at 7.25. A middle tier included ChatGPT (6.42), Gemini (6.33), DeepSeek (6.17), and Copilot (6.00). Lower scores were observed for Claude (5.33), Meta AI (5.08), Pi (5.00), and ClinicalKey AI (4.75), while Dyna AI ranked lowest with a score of 3.75. These differences were statistically significant (χ^2^(11) = 44.19, *p* < 0.0001). These results are summarized in Figure 1.

Based on the pairwise Dunn’s post hoc comparisons (Supplementary Table) higher-scoring models consistently showed statistically significant differences when compared to lower-scoring systems such as Dyna AI, ClinicalKey AI, and Pi. Comparisons among mid-tier models (e.g., ChatGPT, Gemini, DeepSeek, and Copilot) were less consistently significant.

**Figure 2:**
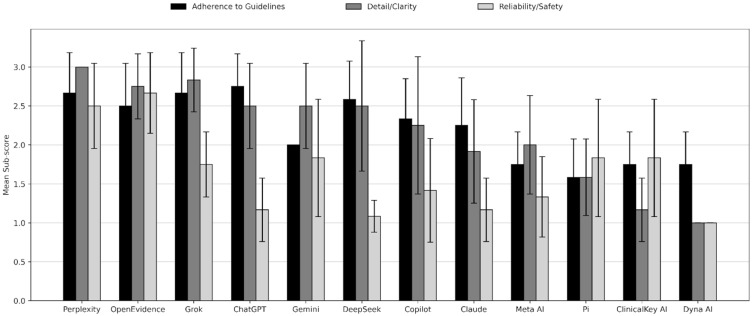
Sub-scores of HTN treatment plan scores across popular LLMs. Each LLM was assigned a score from 1 to 3 by each reviewer across each sub-score domain (adherence to guidelines, detail/clarity, and reliability/safety). Mean sub-scores were calculated and displayed here. Error bars represent standard deviations. Kruskal–Wallis test confirmed statistical significance (*p* < 0.05).

In addition to analyzing the rubric total scores for each LLM, we examined the scores for each domain. Sub-score results are summarized in Figure 2. Across models, adherence to ACC/AHA hypertension guidelines varied but clustered in the mid-to-high range, with ChatGPT achieving the highest adherence score at 2.75 out of 3. Perplexity and Grok followed closely, each scoring 2.67, while DeepSeek (2.58) and OpenEvidence (2.50) also demonstrated strong guideline alignment. Moderate adherence was observed for Copilot (2.33) and Claude (2.25), whereas Gemini scored 2.00. Lower adherence scores were seen for Meta AI, ClinicalKey AI, and Dyna AI, each at 1.75, with Pi scoring the lowest at 1.58. Overall, adherence scores ranged from 1.58 to 2.75, and these differences were statistically significant (χ^2^(11) = 34.42, *p* = 0.0003). Overall, the LLMs provided correct pharmacological recommendations, including advising initiation of two first-line drugs (calcium channel blockers, thiazide diuretics, and angiotensin-converting enzyme inhibitors/angiotensin receptor blockers), lifestyle modifications, follow-up with a physician, and checking pressures at home.

Thoroughness, detail and clarity scores showed a wider spread across models, with Perplexity achieving the highest possible score at 3.00 out of 3. Grok followed with a score of 2.83, and OpenEvidence with 2.75. A second tier of models—including ChatGPT, Gemini, and DeepSeek—each scored 2.50. Copilot demonstrated slightly lower performance at 2.25, while Meta AI scored 2.00 and Claude 1.92. The lowest levels of detail and clarity were observed for Pi (1.58), ClinicalKey AI (1.17), and Dyna AI (1.00). Overall, scores ranged from 1.00 to 3.00, and differences across models were statistically significant (χ^2^(11) = 41.89, *p* < 0.0001).

Reliability and safety scores were generally lower than guideline adherence and detail/clarity, with substantial variability across models. OpenEvidence achieved the highest reliability/safety score at 2.67 out of 3, followed by Perplexity at 2.50. A middle tier included Gemini, Pi, and ClinicalKey AI, each scoring 1.83, while Grok scored 1.75. Lower scores were observed for Copilot (1.42), Meta AI (1.33), and both ChatGPT and Claude at 1.17. DeepSeek scored 1.08, and Dyna AI had the lowest reliability/safety score at 1.00. Overall, scores ranged from 1.00 to 2.67, and differences across models were statistically significant (χ^2^(11) = 36.33, *p* = 0.0001). The references provided by the chatbots can be found in the Supplementary Material. ACC/AHA guidelines were the most frequently cited source of information [17].

## 4. Discussion

We conducted a comparative evaluation of hypertension treatment plans generated by twelve large language models (LLMs), including ChatGPT, Perplexity, OpenEvidence, and others. Responses were scored by blinded reviewers using a rubric defining three major criteria: adherence to ACC/AHA hypertension guidelines, detail and clarity, and safety/reliability (defined as provision of credible sources and advising follow-up with a healthcare professional). Perplexity achieved the highest composite score (8.17/9), followed closely by OpenEvidence (7.92/9), and both demonstrated strong performance in clarity and detail. ChatGPT led in guideline adherence (2.75/3), while Dyna AI scored the lowest overall (3.75/9), particularly underperforming in both detail and safety. Statistical analysis using Kruskal–Wallis test confirmed significant differences across all domains. Post hoc pairwise comparisons of composite scores demonstrated that top-performing models (Perplexity and OpenEvidence) were significantly superior to several lower-performing systems. In contrast, mid-tier models showed fewer significant differences among each other, suggesting comparable performance. Overall scores spanned more than four points (3.75–8.17 out of 9), demonstrating substantial variability in the quality of AI-generated HTN management plans.

Despite generally providing guideline-consistent and detailed treatment plans, many LLMs failed to emphasize the importance of professional medical follow-up or in citing credible sources in the setting of providing stage two hypertension treatment plans. For example, Dyna AI scored only 1/3 in both detail and safety, indicating a lack of sufficient clinical information and user safety measures. The findings highlight that while popular LLMs such as Perplexity and OpenEvidence are capable of generating high-quality clinical content, there remains variability in performance across models, particularly in patient safety guidance. Moreover, chatbots developed specifically for the medical field, such as Dyna AI and ClinicalKey AI, were not found to be necessarily superior to LLMs developed for multi-purpose general use, such as ChatGPT and Perplexity. It is important to note that these findings are specific to the prompt we used and may not be generalizable to other conditions or prompts phrased differently, particularly because only one query was used per system.

Our findings align with several previous studies. Malak and Şahin (2024) [19] evaluated the readability and quality of chatbot-generated information on female urinary incontinence across ten AI platforms, including many assessed in our study (e.g., GPT-4, Gemini, Perplexity, Copilot, Grok, etc.). They found that Gemini achieved the highest mean ratings while Grok had the highest readability. Similar to our study, the researchers found variability across platforms but concluded that, overall, the chatbots could help health professionals in providing medical education to patients [19]. Lorenzi et al. (2024) [20] compared ChatGPT-4 and Gemini Advanced for treatment recommendations in head and neck cancer cases. They found ChatGPT had greater alignment with National Comprehensive Cancer Network (NCCN) guidelines, consistent with our finding that ChatGPT slightly outperformed Gemini in following ACC/AHA guidelines, although in our study, Gemini had a higher composite score than ChatGPT [20]. In a study of LLMs in evidence-based dentistry, Giannakopoulos et al. (2023) found that ChatGPT-4 outperformed Bard, Bing, and ChatGPT-3.5, though all models occasionally produced vague, outdated, or inaccurate information without citing sources [21]. These deficiencies closely resemble the frequent absence of source references in our hypertension study. The consistency of these limitations across specialties points to a fundamental need for transparency and safeguards in LLM outputs. In terms of medical education, Biri et al. (2023) explored the usage of LLMs by medical students and found generally positive attitudes, although students were concerned about being overly reliant on AI and potential inaccurate information provided by LLMs [22]. Ultimately, our findings reinforce previous literature regarding the potential of AI to provide health education while also emphasizing the potential for inaccuracies and lack of transparency.

Importantly, the top two performing models, Perplexity and OpenEvidence, are both RAG systems. Thus, we suggest that architecturally distinct systems (e.g., RAG vs. purely generative models) may produce outputs that are often difficult to distinguish, although referenced citations tend to drive authority independent of clinical accuracy. In other words, evaluators may favor systems with clearly cited references (e.g., Perplexity, OpenEvidence) even if other systems scored higher on adherence to clinical guidelines (e.g., ChatGPT). This may potentially explain why medicine-specific systems were not necessarily superior to general ones; we hypothesize that architectural design (i.e., RAG versus purely generative) may be a more important factor for scoring the responses than the subject of interest targeted by the system. The prompt instruction to “pretend you are a physician” may also have triggered safety guardrails differently across systems, potentially confounding the reliability/safety scores. Specifically, OpenEvidence initially responded that the prompt was out of its scope, so we instead used the following prompt: “Provide a treatment plan for a 50 year old male patient who has just been diagnosed with stage 2 hypertension.” All other systems responded to the original prompt, but it is possible the term “pretend you are a physician” may have influenced the responses, highlighting the importance of using multiple similar prompts in future studies.

Although we attempted to mitigate bias by blinding reviewers and using objective rubrics for scoring, this study is limited by the inherently subjective nature of human reviewers when comparing responses. Moreover, our study only focused on management plans for stage two hypertension. While it is likely that the LLMs would have similar relative quality of responses for other diseases, our results may not be generalizable to rarer diseases or diseases without clear treatment guidelines from professional organizations. It is also true that LLMs are constantly being updated and improved such that future chatbots may provide superior management plans. Unfortunately, we did not record temperature or other randomness controls when the systems were prompted. Furthermore, we only used one prompt for each chatbot, which limits the generalizability of our findings because variations in prompts might influence the generated responses. In future studies, using multiple prompts for each chatbot would improve reproducibility and adjust for intra-model variability. In particular, the sub-scores adherence to guidelines and reliability/safety had Shrout–Fleiss reliability ICC values of 0.42 and 0.46, respectively, indicating fair reliability. Because interpretations of domain-level scores are constrained by these ICC values, the composite scores, which had the highest ICC values, should be considered more reliable than any individual domain. Lastly, our results were obtained prior to publication of the 2025 ACC/AHA joint committee guidelines for management of high blood pressure [23]. While the updated guidelines have not changed regarding the recommended treatment plan for patients with stage two hypertension used in our study, future responses from AI chatbots may rely more on the 2025 guidelines rather than those published in 2017.

Future research on AI-generated treatment plans should emphasize the development and validation of AI systems that include increased citations of reliable sources and emphasize the need to see a healthcare professional for any medical advice. Studies of AI chat bots should also include multiple outputs produced from all systems in order to evaluate both reproducibility and intra-model variability. Additionally, standardized frameworks for evaluating LLM outputs—including interpretability, transparency, and clinician interpretation—will be essential to foster trust and safely integrate AI into clinical workflows, similar to the quality assessment tools discussed by Nasarian et al. in their 2024 systematic review [24]. The creation and adoption of consensus guidelines, such as the FUTURE-AI framework, offer structured pathways to ensure AI systems meet rigorous ethical, regulatory, and performance standards in medical decision-making [25]. Furthermore, investigations into AI-driven treatment optimization, such as personalized therapy simulation, are promising avenues to reduce trial-and-error prescribing and tailor treatment to individual patient profiles [26]. All of these future studies should also take into account the relevance of architectural heterogeneity that we have highlighted here and explicitly compare RAG and purely generative systems.

Rather than being a technical ranking of AI systems, our conclusions, particularly regarding the importance of architectural heterogeneity, support the need for several policy measures to improve transparency and patient safety. First, AI systems providing health-related responses should be required to disclose their knowledge-sourcing mechanisms at the point of interaction, clearly indicating whether answers derive from internal model training, external database retrieval, or a combination of both. Such disclosure would parallel labeling standards used for pharmaceuticals and food products and would allow users to make more informed judgments about informational reliability. Second, regulatory authorities, including agencies such as the U.S. Food and Drug Administration and the European Medicines Agency, should consider requiring standardized warnings when health-related queries are detected on general-purpose AI systems. These warnings should explicitly state that AI responses are not substitutes for professional medical advice and that the presence of citations or confident language does not guarantee clinical accuracy. Finally, independent third-party quality certification for AI systems used in health contexts should be explored. The rubric-based blinded evaluation used in this study demonstrates that systematic assessment of clinical quality is feasible; periodic independent benchmarking with publicly available results could provide transparency similar to that required for pharmaceuticals and medical devices, helping clinicians, patients, and institutions identify AI systems that meet appropriate standards of safety and reliability.

## Data Availability

Data utilized for this study can be found in the Supplementary Material of this article.

## Supplementary Materials

The following supporting information can be downloaded at: https://www.mdpi.com/article/10.3390/jcm15083103/s1, Table S1: Sub-score Averages; Table S2: Raw Data; Table S3: LLM Details; Table S4: Post Hoc Pairwise Testing. References [27,28,29,30,31,32,33,34,35,36,37,38,39,40,41,42] are cited in the Supplementary Materials

## Abbreviations

The following abbreviations are used in this manuscript:

AI	Artificial Intelligence
ACC	American College of Cardiology
AHA	American Heart Association
LLM	Large Language Model
HTN	Hypertension

## References

[1] Elhaddad M., Hamam S. (2024). AI-driven clinical decision support systems: An ongoing pursuit of potential. Cureus.

[2] Hallquist E., Gupta I., Montalbano M., Loukas M. (2025). Applications of artificial intelligence in medical education: A systematic review. Cureus.

[3] Traylor D.O., Kern K.V., Anderson E.E., Henderson R. (2025). Beyond the screen: The impact of generative artificial intelligence (AI) on patient learning and the patient-physician relationship. Cureus.

[4] Patel N., Grewal H., Buddhavarapu V., Dhillon G. (2025). Openevidence: Enhancing medical student clinical rotations with AI but with limitations. Cureus.

[5] MohanaSundaram A., Sathanantham S.T., Ivanov A., Mofatteh M. (2025). DeepSeek’s readiness for medical research and practice: Prospects, bottlenecks, and global regulatory constraints. Ann. Biomed. Eng..

[6] Salman I.M., Ameer O.Z., Khanfar M.A., Hsieh Y.H. (2025). Artificial intelligence in healthcare education: Evaluating the accuracy of ChatGPT, Copilot, and Google Gemini in cardiovascular pharmacology. Front. Med..

[7] Mavrych V., Yaqinuddin A., Bolgova O. (2025). Claude, ChatGPT, Copilot, and Gemini performance versus students in different topics of neuroscience. Adv. Physiol. Educ..

[8] Hurt R.T., Stephenson C.R., Gilman E.A., Aakre C.A., Croghan I.T., Mundi M.S., Ghosh K., Edakkanambeth Varayil J. (2025). The use of an artificial intelligence platform OpenEvidence to augment clinical decision-making for primary care physicians. J. Prim. Care Community Health.

[9] Zhou S., Xu Z., Zhang M., Xu C., Guo Y., Zhan Z., Fang Y., Ding S., Wang J., Xu K. (2025). Large language models for disease diagnosis: A scoping review. Artif. Intell..

[10] Bélisle-Pipon J.C. (2024). Why we need to be careful with LLMs in medicine. Front. Med..

[11] Asgari E., Montaña-Brown N., Dubois M., Khalil S., Balloch J., Yeung J.A., Pimenta D. (2025). A framework to assess clinical safety and hallucination rates of LLMs for medical text summarisation. Digit. Med..

[12] Huo B., Boyle A., Marfo N., Tangamornsuksan W., Steen J.P., McKechnie T., Lee Y., Mayol J., Antoniou S.A., Thirunavukarasu A.J. (2025). Large Language Models for Chatbot Health Advice Studies: A Systematic Review. JAMA Netw. Open.

[13] Chen X., Xiang J., Lu S., Liu Y., He M., Shi D. (2025). Evaluating large language models and agents in healthcare: Key challenges in clinical applications. Intell. Med..

[14] Yang R., Ning Y., Keppo E., Liu M., Hong C., Bitterman D.S., Ong J.C.L., Shu D., Ting W., Liu N. (2025). Retrieval-augmented generation for generative artificial intelligence in health care. npj Health Syst..

[15] Mills K.T., Stefanescu A., He J. (2020). The global epidemiology of hypertension. Nat. Rev. Nephrol..

[16] Arima H., Barzi F., Chalmers J. (2011). Mortality patterns in hypertension. J. Hypertens..

[17] Whelton P.K., Carey R.M., Aronow W.S., Casey D.E., Collins K.J., Himmelfarb C.D., DePalma S.M., Gidding S., Jamerson K.A., Jones D.W. (2018). 2017 ACC/AHA/AAPA/ABC/ACPM/AGS/APhA/ASH/ASPC/NMA/PCNA guideline for the prevention, detection, evaluation, and management of high blood pressure in adults: A report of the American College of Cardiology/American Heart Association Task Force on Clinical Practice Guidelines. Hypertension.

[18] Nasra M., Jaffri R., Pavlin-Premrl D., Kok H.K., Khabaza A., Barras C., Slater L.-A., Yazdabadi A., Moore J., Russell J. (2025). Can artificial intelligence improve patient educational material readability? A systematic review and narrative synthesis. Intern. Med. J..

[19] Malak A., Şahin M.F. (2024). How useful are current chatbots regarding urology patient information? Comparison of the ten most popular chatbots’ responses about female urinary incontinence. J. Med. Syst..

[20] Lorenzi A., Pugliese G., Maniaci A., Lechien J.R., Allevi F., Boscolo-Rizzo P., Vaira L.A., Saibene A.M. (2024). Reliability of large language models for advanced head and neck malignancies management: A comparison between ChatGPT 4 and Gemini Advanced. Eur. Arch. Otorhinolaryngol..

[21] Giannakopoulos K., Kavadella A., Salim A.A., Stamatopoulos V., Kaklamanos E.G. (2023). Evaluation of the performance of generative AI large language models ChatGPT, Google Bard, and Microsoft Bing Chat in supporting evidence-based dentistry: Comparative mixed methods study. J. Med. Internet Res..

[22] Biri S.K., Kumar S., Panigrahi M., Mondal S., Behera J.K., Mondal H. (2023). Assessing the utilization of large language models in medical education: Insights from undergraduate medical students. Cureus.

[23] Jones D.W., Ferdinand K.C., Taler S.J., Johnson H.M., Shimbo L.D., Abdalla M., Altieri M., Bansal N., Bello N.A., Bress A.P. (2025). 2025 AHA/ACC/AANP/AAPA/ABC/ACCP/ACPM/AGS/AMA/ASPC/NMA/PCNA/SGIM guideline for the prevention, detection, evaluation, and management of high blood pressure in adults: A report of the American College of Cardiology/American Heart Association Joint Committee on Clinical Practice Guidelines. Hypertension.

[24] Nasarian E., Alizadehsani R., Acharya U.R., Tsui K.-L. (2024). Designing interpretable ML system to enhance trust in healthcare: A systematic review to proposed responsible clinician-AI-collaboration framework. Inf. Fusion.

[25] Lekadir K., Frangi A.F., Porras A.R., Glocker B., Cintas C., Langlotz C.P., Weicken E., Asselbergs F.W., Prior F., Collins G.S. (2025). FUTURE-AI: International consensus guideline for trustworthy and deployable artificial intelligence in healthcare. BMJ.

[26] Serrano D.R., Luciano F.C., Anaya B.J., Ongoren B., Kara A., Molina G., Ramirez B.I., Sanchez-Guirales S.A., Simon J.A., Tomietto G. (2024). Artificial intelligence (AI) applications in drug discovery and drug delivery: Revolutionizing personalized medicine. Pharmaceutics.

[27] Elsevier (2019). Coronary Disease, Screening and Primary Prevention.

[28] Elsevier (2019). Hypertension.

[29] Heizelman R.J. (2022). Telehealth and hypertension management. Prim. Care Clin. Off. Pract..

[30] Clarke S.L. (2023). Hypertension in adults: Initial evaluation and management. Am. Fam. Physician.

[31] Arnett D.K., Blumenthal R.S., Albert M.A., Buroker A.B., Goldberger Z.D., Hahn E.J., Himmelfarb C.D., Khera A., Lloyd-Jones D., McEvoy J.W. (2019). 2019 ACC/AHA guideline on the primary prevention of cardiovascular disease: A report of the American College of Cardiology/American Heart Association Task Force on Clinical Practice Guidelines. J. Am. Coll. Cardiol..

[32] Taler S.J. (2018). Initial treatment of hypertension. N. Engl. J. Med..

[33] Carey R.M., Moran A.E., Whelton P.K. (2022). Treatment of hypertension: A review. JAMA.

[34] Smith D.K., Lennon R.P., Carlsgaard P.B. (2020). Managing hypertension using combination therapy. Am. Fam. Physician.

[35] https://pmc.ncbi.nlm.nih.gov/articles/PMC8109319/.

[36] https://www.mayoclinic.org/diseases-conditions/high-blood-pressure/diagnosis-treatment/drc-20373417.

[37] https://emedicine.medscape.com/article/241381-treatment.

[38] https://www.ahajournals.org/doi/10.1161/HYPERTENSIONAHA.120.15026.

[39] https://www.healthline.com/health/high-blood-pressure-hypertension.

[40] https://www.nhlbi.nih.gov/health/high-blood-pressure/treatment.

[41] https://www.pbm.va.gov/PBM/AcademicDetailingService/Documents/508/10-1685_HTN_ClinicianGuide_P97122.pdf.

[42] https://www.ama-assn.org/delivering-care/hypertension/patients-can-take-these-steps-lower-their-high-blood-pressure.

