# Supplementary material for: Assessment of Stage Two Hypertension Treatment Plans Written by Generative AI
Source: J Clin Med. 2026 Apr 18;15(8):3103. doi: 10.3390/jcm15083103 (PMC13118107; doi:10.3390/jcm15083103)
Supplement: Supplementary file 1 [file jcm-15-03103-s001.zip › jcm-4184509-supplementary.pdf]

JCM Supplementary Material: Metzger et al.

Table S1: Averages

		Averages							
#	LLM	Adherence to Guidelines (p=0.00125)	Detail/Clarity (p<0.00001)	SD Adherance	SD Detail/Clarity	Reliability/Safety (p<0.00003)	Composite (p<<0.00001)	SD Reliability/Safety	SD Composite
1	**Perplexity**	2.66666667	3	0.51639778	0	2.5	8.16666667	0.54772256	0.75277265
2	**OpenEvidence**	2.5	2.75	0.54772256	0.41833001	2.66666667	7.91666667	0.51639778	0.91742393
3	**ChatGPT**	2.75	2.5	0.41833001	0.54772256	1.16666667	6.41666667	0.40824829	0.40824829
4	**DeepSeek**	2.58333333	2.5	0.49159604	0.83666003	1.08333333	6.16666667	0.20412415	1.16904519
5	**Gemini**	2	2.5	0	0.54772256	1.83333333	6.33333333	0.75277265	1.03279556
6	**Dyna AI**	1.75	1	0.41833001	0	1	3.75	0	0.41833001
7	**Claude**	2.25	1.91666667	0.61237244	0.66458007	1.16666667	5.33333333	0.40824829	1.50554531
8	**ClinicalKey AI**	1.75	1.16666667	0.41833001	0.40824829	1.83333333	4.75	0.75277265	1.08397417
9	**Pi**	1.58333333	1.58333333	0.49159604	0.49159604	1.83333333	5	0.75277265	1.26491106
10	**Meta AI**	1.75	2	0.41833001	0.63245553	1.33333333	5.08333333	0.51639778	1.02062073
11	**Grok**	2.66666667	2.83333333	0.51639778	0.40824829	1.75	7.25	0.41833001	0.98742088
12	**Copilot**	2.33333333	2.25	0.51639778	0.88034084	1.41666667	6	0.66458007	1.26491106

Table S2: Raw Data
Domain 1 (D1) = adherence to clinical guidelines
Domain 2 (D2) = detail/clarity
Domain 3 (D3) = sources/see healthcare professional

		Scorer 1				Scorer 2				Scorer 3				Scorer 4				Scorer 5				Scorer 6			
#	LLM	D1	D2	D3	Total	D1	D2	D3	Total	D1	D2	D3	Total	D1	D2	D3	Total	D1	D2	D3	Total	D1	D2	D3	Total
1	**Perplexity**	2	3	3	8	3	3	2	8	3	3	3	9	3	3	3	9	2	3	2	7	3	3	2	8
2	**OpenEvidence**	2	2.5	3	7.5	3	3	2	8	3	3	3	9	2	2	3	7	2	3	2	7	3	3	3	9
3	**ChatGPT**	2.5	2	1	5.5	3	2	1	6	3	3	2	8	3	2	1	6	2	3	1	6	3	3	1	7
4	**DeepSeek**	2.5	2	1.5	6	3	3	1	7	3	3	1	7	2	1	1	4	2	3	1	6	3	3	1	7
5	**Gemini**	2	2	1	5	2	3	2	7	2	2	3	7	2	3	2	7	2	3	2	7	2	2	1	5
6	**Dyna AI**	1.5	1	1	3.5	2	1	1	4	2	1	1	4	1	1	1	3	2	1	1	4	2	1	1	4
7	**Claude**	1.5	1.5	1	4	2	2	1	5	3	3	2	8	3	2	1	6	2	2	1	5	2	1	1	4
8	**ClinicalKey AI**	1.5	1	1	3.5	2	1	2	5	2	2	2	6	1	1	2	4	2	1	3	6	2	1	1	4
9	**Pi**	1.5	1.5	1	4	1	2	2	5	2	2	3	7	2	1	1	4	1	1	2	4	2	2	2	6
10	**Meta AI**	1.5	2	1	4.5	2	3	1	6	2	2	2	6	2	1	1	4	1	2	1	4	2	2	2	6
11	**Grok**	2	2	1.5	5.5	3	3	1	7	3	3	2	8	3	3	2	8	2	3	2	7	3	3	2	8
12	**Copilot**	2	1.5	2.5	6	2	3	1	6	3	3	2	8	2	1	1	4	2	3	1	6	3	2	1	6

Table S3: LLM Details

	Date prompted	Version	Account tier	Sources retrieved
**ChaGPT (OpenAI)**	June 5, 2025	GPT-4o	Free	"per ACC/AHA guidelines"
**Claude (Anthropic)**	June 5, 2025	Claude 4 Sonnet	Free	N/A
**ClinicalKey AI (Elsevier)**	June 5, 2025	No versions publically available	Free trial	1.Coronary Disease, Screening and Primary Prevention. [Elsevier] Clinical Overview Details<br>2. Hypertension. [Elsevier] Clinical Overview Details<br>3. Telehealth and Hypertension Management. Heizelman RJ. Primary Care: Clinics in Office Practice. 2022;49(4):621-629. doi:10.1016/j.pop.2022.05.003 Journal Article. Article last updated: November 30, 2022 Details

				4. Hypertension, Diagnosis and Initial Treatment. [Elsevier] Clinical Overview
**Copilot (Microsoft)**	June 12, 2025	Copilot Wave 2	Free	"ACC/AHA"
**DeepSeek**	June 5, 2025	DeepSeek-V3-0324	Free	"per ACC/AHA"
**Dyna AI**	June 5, 2025	No versions publically available	Free trial	N/A
**Google Gemini (2.5 Flash)**	June 5, 2025	Gemini 2.5 Flash	Free	N/A
**Grok (X)**	June 5, 2025	Grok 3	Free	"ACC/AHA 2017"
**Meta AI assistant**	June 5, 2025	Llama 4 Maverick	Free	N/A
**OpenEvidence**	June 5, 2025	Version 2.0	Free	Hypertension in Adults: Initial Evaluation and Management. Clarke SL. American Family Physician. 2023;108(4):352-359.<br><br>2017 ACC/AHA/AAPA/ABC/ACPM/AGS/APhA/ASH/ASPC/NMA/PCNA Guideline for the Prevention, Detection, Evaluation, and Management of High Blood Pressure in Adults: A Report of the American College of Cardiology/American Heart Association Task Force on Clinical Practice Guidelines. Whelton PK, Carey RM, Aronow WS, et al. Journal of the American College of Cardiology. 2018;71(19):e127-e248. doi:10.1016/j.jacc.2017.11.006. Leading Journal<br><br>2019 ACC/AHA Guideline on the Primary Prevention of Cardiovascular Disease: A Report of the American College of Cardiology/American Heart Association Task Force on Clinical Practice Guidelines. Arnett DK, Blumenthal RS, Albert MA, et al. Journal of the American College of Cardiology. 2019;74(10):e177-e232. doi:10.1016/j.jacc.2019.03.010. Leading Journal<br><br>Initial Treatment of Hypertension. Taler SJ. The New England Journal of Medicine. 2018;378(7):636-644. doi:10.1056/NEJMcp1613481. Leading Journal

				Treatment of Hypertension: A Review. Carey RM, Moran AE, Whelton PK. Jama. 2022;328(18):1849-1861. doi:10.1001/jama.2022.19590. Leading Journal<br><br>Managing Hypertension Using Combination Therapy. Smith DK, Lennon RP, Carlsgaard PB. American Family Physician. 2020;101(6):341-349.
**Perplexity**	June 5, 2025	Backend model: Sonar (version not available)	Free	https://pmc.ncbi.nlm.nih.gov/articles/PMC8109319/<br><br>https://www.mayoclinic.org/diseases-conditions/high-blood-pressure/diagnosis-treatment/drc-20373417<br><br>https://emedicine.medscape.com/article/241381-treatment<br><br>https://www.ahajournals.org/doi/10.1161/HYPERTENSIONAHA.120.15026<br><br>https://www.healthline.com/health/high-blood-pressure-hypertension<br><br>https://www.nhlbi.nih.gov/health/high-blood-pressure/treatment<br><br>https://www.pbm.va.gov/PBM/AcademicDetailingService/Documents/508/10-1685_HTN_ClinicianGuide_P97122.pdf<br><br>https://www.ama-assn.org/delivering-care/hypertension/patients-can-take-these-steps-lower-their-high-blood-pressure
**Pi**	June 5, 2025	Inflection-2.5	Free	N/A

Table 3: Post-Hoc Pairwise Testing

**Comparisons**	**Adjusted P-Value**
ChatGPT - Claude	0.19234417983057384
ChatGPT - ClinicalKey AI	0.08643485416

Claude - ClinicalKey AI	0.31211882040083294
ChatGPT - Copilot	0.3943263047553228
Claude - Copilot	0.3041434696948499
ClinicalKey AI - Copilot	0.14554286237313377
ChatGPT - DeepSeek	0.43827519047839925
Claude - DeepSeek	0.22725014809874025
ClinicalKey AI - DeepSeek	0.1124470838815002
Copilot - DeepSeek	0.43206090253414753
ChatGPT - Dyna AI	0.013175697961341564
Claude - Dyna AI	0.1141800343939944
ClinicalKey AI - Dyna AI	0.22226956488736652
Copilot - Dyna AI	0.03281755232
DeepSeek - Dyna AI	0.020162941261772194
ChatGPT - Gemini	0.5
Claude - Gemini	0.19681730029174999
ClinicalKey AI - Gemini	0.08975927162
Copilot - Gemini	0.4014958739326923
DeepSeek - Gemini	0.44523193953361195
Dyna AI - Gemini	0.01427367279
ChatGPT - Grok	0.2245975577796894
Claude - Grok	0.05480100698

ClinicalKey AI - Grok	0.015237167071559327
Copilot - Grok	0.15046577442514528
DeepSeek - Grok	0.18580440915558558
Dyna AI - Grok	0.001659300606
Gemini - Grok	0.22948011338359567
ChatGPT - Meta AI	0.145666129
Claude - Meta AI	0.43752179555789755
ClinicalKey AI - Meta AI	0.3933328014981787
Copilot - Meta AI	0.22473879818478226
DeepSeek - Meta AI	0.1837224591
Dyna AI - Meta AI	0.15200513865246468
Gemini - Meta AI	0.14971241034690927
Grok - Meta AI	0.03282294607
ChatGPT - OpenEvidence	0.1129123621629527
Claude - OpenEvidence	0.013337445850588879
ClinicalKey AI - OpenEvidence	0.003847460265
Copilot - OpenEvidence	0.05917632271
DeepSeek - OpenEvidence	0.09050392153
Dyna AI - OpenEvidence	1.575721400589654e-4
Gemini - OpenEvidence	0.11680589189270968
Grok - OpenEvidence	0.3201195425728534
Meta AI - OpenEvidence	0.00817416274

ChatGPT - Perplexity	0.08054714952
Claude - Perplexity	0.009082403044
ClinicalKey AI - Perplexity	0.001788469252
Copilot - Perplexity	0.03545728588
DeepSeek - Perplexity	0.05890132577
Dyna AI - Perplexity	9.179954231285251e-5
Gemini - Perplexity	0.0840491995
Grok - Perplexity	0.23935679242626282
Meta AI - Perplexity	0.004802324346
OpenEvidence - Perplexity	0.43034930710612895
ChatGPT - Pi	0.13939636058796356
Claude - Pi	0.41615065889472047
ClinicalKey AI - Pi	0.41500584537191443
Copilot - Pi	0.21514346769175297
DeepSeek - Pi	0.16287945478281876
Dyna AI - Pi	0.15896051213272086
Gemini - Pi	0.14375249685633743
Grok - Pi	0.027654003680064362
Meta AI - Pi	0.47368163723648066
OpenEvidence - Pi	0.007712284986
Perplexity - Pi	0.004143608915
